# 581. Inhalation Injury Mediates the Link Between Air Pollution and Poor Burn Recovery

**DOI:** 10.1093/jbcr/irag033.354

**Published:** 2026-04-06

**Authors:** Maya Matheny, Erin E Ross, Haig A Yenikomshian, Trevor A Pickering, Connie Valencia, Caitlin M Orton, Colleen M Ryan

**Affiliations:** Keck School of Medicine, University of Southern California, Pasadena, CA; Department of Medicine, Harbor-University of California Los Angeles Medical Center, Los Angeles, CA; Division of Plastic and Reconstructive Surgery, Keck School of Medicine, University of Southern California, Los Angeles, CA; Department of Population and Public Health Sciences, Keck School of Medicine, University of Southern California, Los Angeles, CA; Equity Research Institute, University of Southern California, Los Angeles, CA; Department of Surgery, University of Washington Medicine Regional Burn Center, Harborview Medical Center, Seattle, WA; Massachusetts General Hospital, Harvard Medical School, Boston, MA

## Abstract

**Introduction:**

Chronic air pollution, specifically particulate matter 2.5 (PM2.5), increases the risk and severity of acute respiratory distress syndrome (ARDS), and is most concentrated in lower-income areas. Burn survivors from these communities may be especially vulnerable to respiratory complications of burn injury and inhalation injury (IH). This study aims to investigate the impact of chronic PM2.5 exposure on clinical and respiratory outcomes following acute burn injury.

**Methods:**

This was a retrospective national longitudinal cohort study of adult burn survivors between 2015 and 2021. Recorded home census tract code was used to estimate 3-year pre-burn PM2.5 exposure from the National Environmental Public Health tracking network (CDC). Outcomes included mortality, ventilator days, tracheostomy, hospital length of stay (LOS), and patient-reported outcomes of PROMIS Global Health and employment status at 6 and 12 months. Associations with estimated PM2.5 exposure were assessed using mixed-effects regression with census tract as a random effect, unadjusted and adjusted for age, sex, race/ethnicity, total burn surface area (TBSA), and IH.

**Results:**

A total of 582 adult burn survivors were included, with a mean age of 46.2 (Table 1). In unadjusted models, 3-year PM2.5 exposure was associated with longer LOS (IRR 1.07 per μg/m^3^, 95% CI [1.01 – 1.14], *p*=.016), worse PROMIS Global Health score (β = –0.68, 95% CI [-1.32 – -0.03], *p*=.04), and a decreased likelihood of being employed at follow-up (pooled 6- and 12-months, OR 0.72, 95% CI [0.52 – 0.99], *p*=.04, Table 2a). Adjusted models showed no association of these outcomes with PM2.5 exposure; though PM2.5 exposure was associated with IH (OR = 2.16, 95% CI [1.49 – 3.14], *p*<.001), which was strongly associated with prolonged ventilation (IRR = 1.87, 95% CI [1.14 – 3.06], *p*=.013), tracheostomy (OR = 18.5, 95% CI [8.2 – 41.8], *p*<.001), lower Global Health (β = –1.85, 95% CI [–4.82 - 1.12], *p*=.22), and lower employment (OR = 0.30, 95% CI [0.02 – 4.24], *p*=.373, Table 2b).

**Conclusions:**

Higher PM2.5 exposure was associated with a greater likelihood of IH; IH was, in turn, associated with poorer outcomes, suggesting a potential pathway through which environmental exposures may worsen burn recovery. Further study is needed to elucidate whether there is a causal association of PM2.5 with IH, or if other factors account for the geographic variation observed.

**Applicability of Research to Practice:**

Recognizing the connection between environmental exposures and burn outcomes highlights the importance of preventive policies and community-based strategies in reducing risk among vulnerable populations.

**Funding for the study:**

The contents of this abstract were developed under a grant from the National Institute on Disability, Independent Living, and Rehabilitation Research (NIDILRR grant number 90DPBU0007). NIDILRR is a Center within the Administration for Community Living (ACL), Department of Health and Human Services (HHS). The contents of this manuscript do not necessarily represent the policy of NIDILRR, ACL, or HHS, and you should not assume endorsement by the Federal Government.

